# Pharmacokinetics and Drug Metabolism in Canada: The Current Landscape—A Summary of This Indispensable Special Issue

**DOI:** 10.3390/pharmaceutics10010013

**Published:** 2018-01-16

**Authors:** Neal M. Davies, Kishor M. Wasan

**Affiliations:** 1 Faculty of Pharmacy and Pharmaceutical Sciences, University of Alberta, Edmonton, AB T6G 2R3, Canada; 2 College of Pharmacy and Nutrition, University of Saskatchewan, Saskatoon, SK S7N 2Z4, Canada

Canadian Pharmaceutical Scientists have a rich history of groundbreaking research in pharmacokinetics and drug metabolism undertaken primarily throughout its Pharmacy Faculties and within the Pharmaceutical and Biotechnology industry. The principles of drug absorption, distribution, metabolism, and excretion (ADME) is the foundational basis of rationale drug-design, and principled pharmacotherapy. The study of ADME and its descriptive quantitative analysis is the basis of pharmacokinetics. Pharmacokinetics is fundamental in the development of a new chemical entity into a marketable product and is essential in understanding the bioavailability, bioequivalence, and biosimilarities of drugs. Pharmacokinetics and drug metabolism and development studies facilitate an understanding of organ-based functionality. Population pharmacokinetic variability and the modeling of drug concentrations has significant utility in translating individual response in a target patient population underlying advances in precision health.

This special issue serves to highlight and capture the contemporary progress and current landscape of pharmacokinetics and drug metabolism within the prevailing Canadian context and the impact this pharmaceutical sciences research has had on an international scientific community.

This special issue presents a series of review articles highlighting a summary of the research that investigators from across the country have completed, thus making meaningful and significant contributions to the field. El-Kadi and colleagues from the University of Alberta summarized the clinical implications and impact of 20-hydroxyeicosatetraenoic acid in the kidney, liver, lung, and brain as a potential therapeutic target for many diseases [1]. Liu and Coughtrie from the University of British Columbia revised their paper about the latency of uridine diphosphate-glucuronosyltransferases (UGTs) and how the endoplasmic reticulum membrane influences their function [2].

Mahmoud and Shen from the University of Alberta discusses augmented renal clearance (ARC) as a manifestation of enhanced renal function seen in critically ill patients and show that the use of regular unadjusted doses of renally eliminated drugs in patients with ARC might lead to therapy failure [3].

Lin and Wong from the University of British Columbia focus on the development of orally absorbed physiologically based pharmacokinetic (PBPK) models and briefly discuss the major applications of these models in the pharmaceutical industry [4]. Kiang from the University of Alberta and colleagues from University of British Columbia provide a qualitative review on (1) the principles of therapeutic drug monitoring (TDM); (2) alternative matrices for TDM; (3) current evidence supporting the use of interstitial fluid (ISF) for TDM in clinical models; (4) the use of microneedle technologies, which is potentially minimally invasive and pain-free, for the collection of ISF; and (5) future directions [5].

In addition, this special issue has published a series of new leading edge translational research articles demonstrating the continued vibrant collaborative activity of our pharmaceutical sciences community. Edginton with Canadian colleagues from McMaster and Waterloo explored different weight metrics including lean body weight, ideal body weight, and adjusted body weight to determine an alternative dosing strategy that is both safe and resource-efficient in normal and overweight/obese adult patients [6]. Lakowski and colleagues from University of Manitoba and Alberta identified the metabolism, excretion, antioxidant, anti-inflammatory, and anticancer properties of curcuminoids and determined disposition in rodents [7,8]. Simard and colleagues from Université Laval set out to determine if altered protein expression of cardiac and hepatic drug metabolizing enzymes in a mouse model of Type II diabetes lead to the onset and development of cardiovascular disease [9]. Leung, Turgeon, and Michaud from the Université de Montréal presented a study of statin- and loratadine-induced muscle pain mechanisms using human skeletal muscle cells [10]. The same group lead by Michaud and colleagues investigated the specific modulation of cyp2c and cyp3a mRNA levels and activities via diet-induced obesity in mice and the impact of Type II diabetes on drug metabolizing enzymes in liver and extra-hepatic tissues [11].

Davies, Lobenberg, Burczynski, and colleagues from the University of Manitoba and Alberta with international collaborators completed a pharmacokinetic analysis of an oral multicomponent joint dietary supplement in dogs as well as a pharmacokinetic and toxicodynamic characterization of a new doxorubicin derivative with reported lymphatic delivery [12]. Sitar and colleagues from the University of Manitoba and University of Toronto investigated a new theophylline metabolite, theophylline-7β-d-ribofuranoside (theonosine), generated in human and animal lung tissue [13].

Brocks and colleagues from the University of Alberta reported on the development of a selective and sensitive high-performance liquid chromatographic method for the determination of lidocaine in human serum and its application to clinical pharmacokinetics [14]. Foster and colleagues based in Alberta, Canada, and ContraVir Pharmaceuticals Inc., located in Edison, New Jersey, in the United States discussed the in vitro Phase I metabolism of CRV431, a new oral drug candidate for chronic hepatitis B [15].

Piquette-Miller and Gahir from the University of Toronto and Reata Pharmaceuticals, respectively, discussed the role of the PXR genotype and transporter expression in the placental transport of lopinavir in mice [16].

Ellen Wasan and colleagues from the University of Saskatchewan provided an overview of the chitosan-based nanoparticles for various non-parenteral applications and highlighted current research, including sustained release and mucoadhesive chitosan dosage forms that can alter input and pharmacokinetics and targeting [17]. Collier from the University of British Columbia with a cross boarder collaboration with colleagues from Hawaii, USA, unraveled the regulation of hepatic UGT2B15 via methylation in adults of Asian descent [18]. Finally, in a tri-nation international collaboration (Canada, USA, and Qatar) with its roots at the University of Manitoba, Faculty of Pharmacy in Winnipeg, Rachid, Rawas-Qalaji, and Simons extended their investigations towards the development of a novel sublingual epinephrine tablet formulation for anaphylaxis for potential pediatric use in a pre-clinical study [19].

Taken together, these papers represent only a fraction of the important and contemporary research in pharmaceutical sciences across Canada and represent the breadth and depth of work carried out in our fine world class institutions by preeminent pharmaceutical scientists in the areas of pharmacokinetics and drug metabolism. Given a recent review of the structure of funding agencies and the recommended improvements to governance and coordination in Canada, strengthening pharmaceutical research and support of early career pharmaceutical scientists is prudent. Canada’s future as a knowledge-based economy is strengthened by the pharmaceutical research conducted across the nation and the acquisition of pharmaceutical knowledge, and the success of governments’ strategies on technology, innovation, and health sciences could be further enhanced through increased support of leading-edge pharmaceutical investigations highlighted here. Canada, both in research and economically, is more competitive and better positioned on the world stage because of its thriving and vibrant pharmaceutical research community, which this special issue aims to demonstrate.
